# HIV, antiretroviral therapy and non‐communicable diseases in sub‐Saharan Africa: empirical evidence from 44 countries over the period 2000 to 2016

**DOI:** 10.1002/jia2.25364

**Published:** 2019-07-28

**Authors:** Lelani Coetzee, Lisa Bogler, Jan‐Walter De Neve, Till Bärnighausen, Pascal Geldsetzer, Sebastian Vollmer

**Affiliations:** ^1^ University of Pretoria Pretoria South Africa; ^2^ Department of Economics and Centre for Modern Indian Studies University of Goettingen Goettingen Germany; ^3^ Heidelberg Institute of Global Health Medical Faculty and University Hospital Heidelberg Germany; ^4^ Harvard T.H. Chan School of Public Health Boston MA USA

**Keywords:** HIV, antiretroviral therapy, non‐communicable diseases, ageing, sub‐Saharan Africa, cardiovascular risk factors

## Abstract

**Introduction:**

The HIV‐infected population is growing due to the increased accessibility of antiretroviral therapy (ART) that extends the lifespan of people living with HIV (PLHIV). We aimed to assess whether national HIV prevalence and ART use are associated with an increased prevalence of cardiovascular risk factors.

**Methods:**

Using country‐level data, we analysed the effect of HIV prevalence and use of ART on cardiovascular risk factors in 44 countries in sub‐Saharan Africa between 2000 and 2016. We used fixed‐effects estimation to quantify the effect of HIV and ART on the prevalence of diabetes, mean body mass index, the prevalence of overweight, obesity and hypertension, and mean systolic blood pressure. The models were adjusted for calendar time, the age structure of the population, income and education.

**Results:**

Diabetes prevalence among PLHIV was 5.8 percentage points higher (95% confidence interval (CI) 1.8 pp to 9.8 pp) compared to individuals without HIV. People receiving ART had a 4.6 percentage point higher prevalence (95% CI 2.6 pp to 6.6 pp). The prevalence of obesity was increased by 14.7 percentage points (95% CI 2.5 pp to 26.9 pp) for PLHIV. Receiving ART was associated with an increased obesity prevalence by 14.0 percentage points (95% CI 4.8 pp to 23.2 pp), whereas it had no significant association with the prevalence of overweight. The population aged 40 to 59 had a significantly higher prevalence of diabetes, overweight and obesity. HIV prevalence and ART use had no significant association with the prevalence of hypertension.

**Conclusions:**

An ageing HIV‐infected population on ART is associated with a significant increase in the prevalence of diabetes and obesity in sub‐Saharan Africa. The increasing prevalence of these cardiovascular risk factors emphasizes the need for comprehensive healthcare programmes that screen and treat both HIV and non‐communicable diseases to decrease the associated morbidity and mortality rates.

## 1 Introduction

The increasing prevalence of non‐communicable diseases (NCDs) and number of people living with HIV (PLHIV) pose great challenges to global public health. NCDs account for approximately 70% of global deaths per annum 1, whereas the human immunodeficiency virus (HIV) is a communicable disease that has caused approximately 35 million deaths since the epidemic started 2. Globally, sub‐Saharan Africa is most severely affected by HIV, accounting for approximately two‐thirds of the worldwide population living with HIV 3, and it is the region with the highest predicted increase in rates of cardiovascular risk factors 4. The World Health Organization (WHO) estimates that the region will have the largest increase in deaths caused by NCDs over the next decade 5.

Antiretroviral therapy (ART) has turned the once fatal HIV infection into a manageable illness that resembles a chronic disease. While public availability of ART in Africa was very low prior to 2000 6, three events in 2003 dramatically increased the availability of ART in less developed regions in subsequent years 7. First, there was a breakthrough in generic antiretroviral treatments that reduced the number of pills per day and the costs of treatment. Second, the United States established the *Presidential Emergency Plan for AIDS Relief* to financially contribute to the worldwide treatment of HIV 8. Third, the WHO launched the “3 by 5”‐programme that aimed to treat three million HIV‐positive individuals by 2005 9. The number of HIV‐related deaths and new infections per annum has consequently decreased in sub‐Saharan Africa, resulting in an ageing HIV‐infected population 10, 11, 12. A larger ageing HIV‐infected population is likely to increase the prevalence of age‐related NCDs, contributing to NCDs becoming the leading cause of deaths in the region by 2030 5. An ageing HIV‐infected population receiving ART increases the prevalence of NCDs through three pathways 13, 14. First, HIV infection itself may increase the risk of developing NCDs 15, 16, 17, 18. Second, some antiretroviral drugs (particularly the older agents that are generally used in Africa) are thought to have side effects that increase cardiovascular risk factors 19, 20, 21, 22, 23 and the risk of comorbid disease 19, 20. Third, the increased life expectancy due to ART means that PLHIV reach the age with an increased risk of developing age‐related NCDs 24, 25, 26.

There is some evidence from high‐income countries that HIV and ART may increase the prevalence of NCDs 15, 16, 17, 19, 20, 27. However, it is crucial to examine this question more closely in HIV endemic settings, such as sub‐Saharan Africa 28, where it is a question of high importance to population health. In addition, the evidence from high‐income countries cannot easily be extrapolated to sub‐Saharan Africa because the epidemic in high‐income countries is largely restricted to key populations who likely differ in important ways from the general population in sub‐Saharan Africa. Several studies have investigated the association between HIV and NCDs in individual African countries 29, 30, 31, 32, but were limited to only certain regions of single countries using short time spans. One systematic review and meta‐analysis of the association between ART use and hypertension and diabetes mellitus in sub‐Saharan Africa 33, based on 20 cross‐sectional studies, found that ART is associated with dyslipidaemia, but not with hypertension and diabetes. In this study, we go further and conduct a panel analysis to investigate whether national HIV prevalence and ART use are associated with cardiovascular risk factors in 44 countries in sub‐Saharan Africa over the time period 2000 to 2016. We also differentiate between the three pathways through which an ageing HIV‐infected population may increase the prevalence of NCDs.

## 2 Methods

### 2.1 Data sources

The data for the NCD outcome variables (prevalence of diabetes, mean body mass index (BMI), prevalence of overweight and obesity, mean systolic blood pressure and prevalence of hypertension) were taken from the NCD Risk Factor Collaboration (NCD RisC). NCD RisC is a network of health scientists, both researchers and practitioners, that provide yearly aggregate data on NCD risk factors for 200 territories under coordination of the WHO Collaborating Centre on NCD Surveillance and Epidemiology 34. These data are compiled from multiple sources, including more than 2.500 population‐based surveys such as Demographic and Health Surveys from about 200 countries, epidemiological studies with anonymized individual record data, a review of published articles, and unpublished data. NCD RisC re‐analyses the data, converts them to comparable metrics according to a common protocol and regularly updates them with new sources. The surveys are representative of national or subnational populations and potential bias from sources that are not representative of national populations is accounted for. Bayesian hierarchical models are used to estimate missing data by age group, sex and country. Methods of data collection and compilation by NCD RisC are described in detail elsewhere 35, 36, 37, 38.

Yearly data on the percentage of PLHIV, meaning the prevalence of HIV, and the percentage of PLHIV that receive ART were taken from the Health Nutrition and Population Statistics database of the World Bank, which sources the data from UNAIDS estimates 39. We used data on real gross domestic product (GDP) per capita in US dollars (base year 2010) from the World Development Indicators database of the World Bank 40, gross secondary school enrolment ratios from the Education Statistics database of the World Bank 41, and the percentage of the population aged 20 to 39, 40 to 59 and 60 + from the United Nations 42. Data from these different sources were then merged by country and year.

### 2.2 Outcome measures, exposure and control variables

We analysed six outcomes from the NCD RisC data, namely prevalence of diabetes, BMI, prevalence of overweight and obesity, mean systolic blood pressure and prevalence of hypertension. The NCD RisC outcome variables are disaggregated by sex. We used the average value of each corresponding male and female observation per year to allow for our aggregate analysis. In the NCD RisC data, diabetes is defined as fasting plasma glucose of 7.0 mmol/L or higher, or history of diagnosis with diabetes, or use of insulin or oral hypoglycaemic drugs 35. Overweight is defined by a BMI greater or equal to 25 kg/m^2^, while obesity is defined by a BMI greater or equal to 30 kg/m^2^ 38. Hypertension, or raised blood pressure, is defined as systolic blood pressure of 140 mm Hg or higher or diastolic blood pressure of 90 mm Hg or higher 37.

Our key exposures were national HIV prevalence and the proportion of people receiving ART to the entire population. We calculated the latter by multiplying the prevalence of HIV with the percentage of PLHIV who receive ART. The data on the percentage of PLHIV who receive ART were only available from 2000 onward. We therefore restricted the sample to the years 2000 to 2016. This is justified since the history of ART indicates that the public availability of ART in Africa was very low prior to 2000, even until 2002 6, and our data showed zero for all the countries in the sample for the first few years after 2000.

We used income (natural log of real GDP per capita), education (gross secondary school enrolment ratio) and the age structure of the population as covariates in the analysis, while controlling for time. The data on gross secondary school enrolment ratios contained many missing values, and the age variables were only recorded every five years. To increase the sample size and yield more precise estimates, we employed linear interpolation on both of these variables.

### 2.3 Statistical analysis

We specified a series of linear regression models for prevalence of diabetes, mean BMI, prevalence of overweight and obesity, mean systolic blood pressure and prevalence of hypertension as outcome variables. The analysis included yearly data from 44 sub‐Saharan African countries from 2000 to 2016. Each regression model was adjusted for country fixed effects and controlled for time by adding a linear time trend as additional independent variable. Control variables were added in a step‐wise procedure. We used robust standard errors clustered at the country level and reported the clustering‐adjusted coefficients and confidence intervals. The approach can be summarized as follows:

$$Y_{\mathrm{ct}}=\beta_{1}HIV_{\mathrm{ct}}+\beta_{2}ART_{\mathrm{ct}}+\beta_{4}X_{\mathrm{ct}}+\beta_{4}Year+\gamma_{c}+\epsilon_{\mathrm{ct}}$$

where the dependent variable *Y*_ct_ was the outcome (e.g. diabetes prevalence) in country *c* in year *t*, HIV_ct_ and ART_ct_ were the prevalence of HIV and ART use in country *c* in year *t* and *X*_ct_ was a vector of control variables. Year was the linear time trend, increasing by one for each year, and *Y*_c_ captured country fixed effects, resulting in country‐specific constants. The coefficients of interest were *β*_1_ and *β*_2_, which showed the association of HIV and ART use prevalence with the respective outcome.

As a sensitivity analysis, we re‐estimated the equations for all outcome variables using two alternatives to control for time, namely by substituting the linear time trend with year dummies and by adding a quadratic time indicator. In addition, we systematically excluded the top and bottom 3 percentiles of the outcome variables in the sample. Lastly, we re‐estimated the model with all control variables by excluding each country once in order to identify if any one outlier country drove the results. All statistical analyses were done using Stata 14.

### 2.4 Role of the funding source

The funders of the study had no role in study design, data collection, analysis, interpretation of data, writing of the report or in the decision to submit for publication.

## 3 Results

Table 1 shows the prevalence of HIV, ART use and all outcomes in the most recent year, separately by country. HIV prevalence ranged from 0.1% in Comoros to 27.2% in Eswatini. The prevalence of use of ART ranged from 0.01% in Madagascar to 21.5% in Eswatini, the country with the highest HIV prevalence in 2016. The highest prevalence of diabetes was found in Mauritius (13.0%), followed by South Africa (11.1%) and the Seychelles (10.4%). Overweight and obesity were most prevalent in South Africa (54.6% and 28.5% respectively) and Botswana (44.5% and 19.5%), whereas the prevalence of hypertension was highest in Niger (33.6%), followed closely by and Somalia (32.9%) and Chad (32.7%).

**Table 1:** Sample characteristics

Country	HIV (%)	ART (%)	Diabetes (%)	BMI (kg/m^2^)	Overweight (%)	Obesity (%)	Hypertension (%)	Systolic BP (mm Hg)
Angola	1.90	0.42	8.17	23.41	28.20	8.42	29.59	129.62
Burundi	1.10	0.67	4.17	21.73	22.83	5.60	29.19	129.70
Benin	1.00	0.57	7.19	23.62	30.29	9.87	27.60	126.61
Burkina Faso	0.80	0.48	6.41	22.20	23.50	5.60	32.28	126.56
Botswana	21.90	18.18	8.52	24.47	44.55	19.50	29.37	130.89
Central African Republic	4.00	0.96	7.79	22.93	26.74	7.64	31.09	130.15
Cote d'Ivoire	2.70	1.11	6.83	23.86	33.16	10.95	27.31	130.37
Cameroon	3.80	1.41	6.71	24.44	34.60	11.78	24.76	128.30
Congo, Dem. Rep.	0.70	0.29	6.15	22.08	25.94	6.93	28.42	128.33
Congo, Rep.	3.10	0.71	7.67	23.31	31.95	9.94	26.17	129.23
Comoros	0.10	0.04	7.94	24.56	27.94	8.08	27.80	129.66
Cabo Verde	0.80	0.46	8.54	24.61	35.68	12.11	29.31	132.08
Eritrea	0.60	0.35	5.51	20.75	22.33	5.02	28.85	123.05
Ethiopia	1.10	0.65	5.41	20.62	21.52	4.62	30.25	127.78
Gabon	3.60	2.27	10.00	25.42	41.52	15.60	25.39	127.94
Ghana	1.60	0.54	6.50	23.99	32.70	11.04	23.70	124.29
Guinea	1.50	0.52	6.40	22.98	27.45	7.94	30.17	132.60
Gambia, The	1.70	0.51	8.69	24.11	32.85	10.67	29.18	131.52
Guinea‐Bissau	3.10	1.02	7.10	23.51	30.83	9.76	30.21	129.46
Equatorial Guinea	6.20	2.67	9.34	24.21	28.09	8.56	28.42	129.38
Kenya	5.40	3.46	5.99	23.41	26.12	7.29	26.57	129.11
Liberia	1.60	0.30	7.70	24.19	31.88	10.28	28.24	129.92
Lesotho	25.00	13.25	8.58	24.97	38.68	16.30	28.42	128.15
Madagascar	0.20	0.01	5.55	21.36	24.57	5.50	28.00	129.98
Mali	1.00	0.35	7.37	23.09	28.77	8.84	32.40	126.93
Mozambique	12.30	6.64	6.37	22.78	26.75	7.21	28.86	132.96
Mauritania	0.50	0.12	8.93	25.06	35.37	13.09	31.57	129.59
Malawi	9.20	6.07	6.31	22.93	24.02	5.93	28.70	133.64
Namibia	13.80	8.83	7.43	24.30	40.94	17.16	28.29	127.36
Niger	0.40	0.13	5.63	21.92	22.96	5.84	33.55	134.72
Nigeria	2.90	0.87	6.15	23.34	29.99	9.24	23.85	123.45
Rwanda	3.10	2.48	4.41	22.02	25.44	5.87	26.53	126.94
Sudan	0.20	0.02	8.89	24.71	28.94	8.44	30.13	128.86
Senegal	0.40	0.21	7.41	23.02	28.71	8.82	30.06	129.54
Sierra Leone	1.70	0.44	6.87	22.75	28.56	8.93	30.21	130.73
Somalia	0.40	0.04	6.84	23.26	29.14	8.47	32.85	130.76
Eswatini	27.20	21.49	9.56	26.91	38.72	16.43	29.47	129.03
Chad	1.30	0.51	7.02	22.01	23.74	6.28	32.75	130.08
Togo	2.10	1.07	7.14	23.62	28.70	8.54	28.72	127.47
Tanzania	4.70	2.91	6.08	23.28	28.53	8.69	27.15	131.49
Uganda	6.50	4.36	4.57	22.48	23.14	5.46	27.23	127.38
South Africa	18.90	10.58	11.15	27.35	54.58	28.49	26.71	125.05
Zambia	12.40	8.06	6.57	22.64	28.71	8.36	27.05	131.08
Zimbabwe	13.50	10.13	7.04	23.87	38.78	15.63	28.03	128.15

Descriptive statistics of main explanatory variables and outcome variables in the last available year. 2016 for HIV prevalence, ART prevalence, mean BMI, prevalence of overweight and obesity; 2015 for systolic blood pressure and hypertension (raised blood pressure); 2014 for diabetes.

Table 2 shows the association of HIV and ART prevalence with all six outcomes. All regressions controlled for income, education and the age structure of the population. Regressions on the prevalence of diabetes, hypertension and mean systolic blood pressure additionally controlled for mean BMI. Results of stepwise addition of control variables can be found in the appendix. Coefficients on the main explanatory variables, HIV prevalence and use of ART, should be read as the effect on the outcome, if HIV prevalence or use of ART changed from 0% to 100%. In reality, this does not occur on a country level. We therefore interpreted the findings as a comparison in the prevalence of outcomes between PLHIV and those not infected with HIV or between PLHIV receiving ART and PLHIV not receiving ART respectively. The results in the first column of Table 2 show that PLHIV had an increased prevalence of diabetes by 5.8 percentage points (95% CI 1.8 pp to 9.8 pp) at a 1% significance level, compared with the population without HIV. The population on ART had an increased prevalence of diabetes by 4.6 percentage points (95% CI 2.6 pp to 6.6 pp) at a 1% significance level, whereas a one‐unit increase in mean BMI was associated with an increased prevalence of diabetes by 0.9 percentage points (95% CI 0.6 pp to 1.2 pp) also at a 1% significance level. Individuals aged 40 to 59 formed the only age group for whom the prevalence of diabetes was significantly increased.

**Table 2:** Regression results of main specification

Variables	Diabetes prevalence	Mean BMI	Overweight prevalence	Obesity prevalence	Hypertension prevalence	Mean systolic blood pressure
HIV	0.058 (0.018 to 0.098)***	0.665 (−1.813 to 3.142)	0.111 (−0.023 to 0.245)	0.147 (0.025 to 0.269)**	−0.096 (−0.305 to 0.112)	−17.385 (−41.736 to 6.965)
ART	0.046 (0.026 to 0.066)***	1.229 (−0.558 to 3.016)	0.073 (−0.037 to 0.182)	0.140 (0.048 to 0.232)***	−0.090 (−0.286 to 0.106)	−8.324 (−24.783 to 8.134)
BMI	0.009 (0.006 to 0.012)***				0.003 (−0.019 to 0.024)	0.425 (−1.336 to 2.186)
Education	−0.012 (−0.021 to −0.003)***	0.298 (−0.231 to 0.828)	0.009 (−0.014 to 0.032)	−0.003 (−0.023 to 0.017)	−0.006 (−0.049 to 0.037)	−0.085 (−3.868 to 3.697)
Income	0.000 (−0.002 to 0.002)	−0.077 (−0.327 to 0.172)	−0.001 (−0.011 to 0.009)	−0.005 (−0.013 to 0.004)	0.009 (−0.010 to 0.028)	0.694 (−1.096 to 2.483)
Age 20 to 39	−0.000 (−0.034 to 0.033)	−2.067 (−4.343 to 0.209)*	0.012 (−0.148 to 0.172)	−0.003 (−0.144 to 0.138)	0.150 (−0.183 to 0.482)	11.038 (−17.281 to 39.356)
Age 40 to 59	0.051 (0.009 to 0.094)**	3.951 (0.223 to 7.679)**	0.230 (0.019 to 0.440)**	0.234 (0.056 to 0.413)**	−0.480 (−0.797 to −0.162)***	−32.407 (−54.985 to −9.829)***
Age 60+	−0.130 (−0.298 to 0.037)	−2.709 (−14.449 to 9.030)	0.284 (−0.343 to 0.911)	0.547 (0.034 to 1.061)**	−0.612 (−1.747 to 0.523)	−23.994 (−120.622 to 72.635)
Year	0.001 (0.001 to 0.001)***	0.074 (0.062 to 0.086)***	0.005 (0.005 to 0.006)***	0.003 (0.002 to 0.003)***	−0.001 (−0.003 to 0.001)	−0.028 (−0.182 to 0.126)
Constant	−1.726 (−2.210 to −1.242)***	−124.831 (−148.020 to −101.642)***	−10.246 (−11.217 to −9.274)***	−5.248 (−6.134 to −0.363) ***	1.624 (−1.645 to 4.894)	174.603 (−94.830 to 444.037)
Observations	559	578	578	578	578	578
Number of countries	42	42	42	42	42	42
Adjusted R_2_	0.973	0.942	0.981	0.951	0.254	0.181

Results of linear regressions; 95% confidence intervals in brackets; *p <* *0.1, **p <* *0.05, ***p <* *0.01; all models adjusted for fixed effects; outcome variables: diabetes prevalence in percent; country‐level mean BMI (kg/m2); overweight as prevalence of BMI ≥ 25 kg/m2 in percent; overweight as prevalence of BMI ≥ 30 kg/m2 in percent; prevalence of raised blood pressure in percent; country‐level mean systolic blood pressure (mm Hg).

The second column of Table 2 presents results for the outcome mean BMI. We did not find robust associations of HIV and ART with mean BMI. This changed when we categorised mean BMI into overweight and obesity. While the proportion of HIV‐infected individuals was significantly associated with the prevalence of overweight in all other specifications (see Table SB3 in the appendix) this association did not reach conventional levels of significance when controlling for age structure (see column 3 of Table 2). The proportion of people receiving ART was not significantly associated with the prevalence of overweight in most of these specifications. The population aged 40 to 59 was more likely to be overweight by 23.0 percentage points (95% CI 19 pp to 44 pp) at a 5% significance level.

Column 4 of Table 2 shows a stronger association of HIV prevalence with the prevalence of obesity. For this outcome, the use of ART was shown to have a highly significant association. PLHIV had an increased obesity prevalence by 14.7 percentage points (95% CI 2.5 pp to 26.9 pp) at a 5% significance level, compared to people who are HIV negative. Individuals on ART had an increased prevalence of obesity of 14.0 percentage points (95% CI 4.8 pp to 23.2 pp) at a 1% significance level. The population aged 40 to 59 and older than 60 were more likely to be obese by 23.4 percentage points (95% CI 5.6 pp to 41.3 pp) and 54.7 percentage points (95% CI 3.4 pp to 106.1 pp) respectively, both at a 5% significance level. PLHIV and those that receive ART did not have an increased prevalence of hypertension or an increased mean systolic blood pressure, as shown in the last two columns of Table 2.

Data constraints decreased the sample sizes for several regressions. The data on prevalence of diabetes were only available until 2014, meaning the sample for this outcome variable consisted of 44 countries over a time span of 14 years. For the outcomes hypertension and mean systolic blood pressure, data were available until 2015, while for the outcomes mean BMI, overweight and obesity, data were available over the complete time span until 2016. The missing values of the education variable that could not be estimated by extrapolating the data further reduced the number of observations in the regressions that include all confounders, as Somalia and Zambia were dropped. All notes regarding the data are summarized in the appendix.

The results of the sensitivity analysis are shown in the appendix. We repeated the regression analyses using two alternatives for capturing time trends. First, we included year dummies for 2000 to 2016 instead of the linear time trend. As a second alternative, we included a quadratic time term in addition to the linear time indicator in order to capture any potential nonlinear time trends. Neither of these alternatives led to noteworthy changes in the size or significance of the coefficients of interest and the coefficients of the quadratic time term were very close to zero.

In a further robustness analysis, we excluded the top and bottom 3 percentiles of all outcome variables in the sample. This led to South Africa being dropped completely from the regressions with the prevalence of overweight and obesity as outcomes. This trimming slightly changed results for the outcome obesity. The association of ART use with obesity prevalence still appeared as significant, suggesting an increase in the prevalence of obesity, while the association of HIV prevalence with obesity prevalence remained positive but lost significance. Results for the other outcomes were robust to the trimming exercise.

Lastly, we re‐ran the regressions of the main specification using the linear time trend, while excluding each country from the sample once (results not shown). We did not find that any country represented an overly influential outlier.

## 4 Discussion

Using data on the proportion of PLHIV and the proportion of people receiving ART in sub‐Saharan Africa, our results show that national HIV prevalence and ART use are associated with increased cardiovascular risk factors – specifically, the prevalence of diabetes and obesity. To the best of our knowledge, this is the first paper to conduct a panel analysis with several NCD risk factors as outcomes for nearly all countries in sub‐Saharan Africa, using the longest time span allowed by data availability for the region.

The results suggest that all three pathways contribute to the association of the HIV infection with the prevalence of NCDs: The HIV infection itself, the side effects of receiving ART, and the development of age‐related NCDs in an ageing HIV‐infected population. The prevalence of HIV and the prevalence of ART use are associated with a significantly increased prevalence of diabetes and obesity, while an ageing population is associated with a significantly increased prevalence of diabetes, overweight and obesity. The population aged 40 to 59 is more likely to be overweight and obese and has a higher prevalence of diabetes. In addition, the population aged 60 and older has a higher prevalence of obesity.

The results show that diabetes is the cardiovascular risk factor in sub‐Saharan Africa that is most prominently affected by the prevalence of HIV and ART in the region, since the absolute change in prevalence is highest for diabetes. This finding is consistent with the known side effects of ART, including increased insulin resistance and changes in lipid levels 19, 20. Furthermore, the use of ART is associated with increased prevalence of obesity, which is a well‐established risk factor for of type‐2 diabetes. The insignificant association of HIV and ART with the prevalence of hypertension highlights the uncertainty whether the infection or the use of ART itself, or its possible interaction with other medication, independently affect the prevalence of hypertension 43.

This study has several limitations. The empirical specification controls for country specific time‐invariant factors as well as general time trends that are common for all countries. In addition, we controlled for a range of time varying covariates. However, the results were biased in case we missed any unobserved time varying covariates that are both correlated with HIV and/or ART and our outcome variables. We can therefore interpret the results as causal only under the assumption that there are no further unobserved factors that could confound the relationship between HIV and ART and our outcome variables after including the country fixed effects and covariates. Regarding data quality, there is uncertainty around the year‐by‐year changes in the NCD risk factor prevalence, HIV prevalence, and ART use. Given that there are incomplete data on diabetes and hypertension in the countries that are part of our sample, part of the year‐to‐year changes are based on modelling assumptions. However, these are the best data available to the authors for the purposes of this analysis.

Moreover, while we distinguished between the different pathways through which HIV infection increases the prevalence of NCDs by controlling for age groups, this is only a crude way to do so. We find evidence that all three pathways seem to be relevant, but we did not measure their relative contribution to the overall effect. Lastly, in this study, we focused on sub‐Saharan Africa. Given the comparatively high rates of HIV prevalence in some sub‐Saharan African countries, it is questionable to what extent these findings can be applied to other world regions.

## 5 Conclusions

In summary, an ageing HIV‐infected population that receives ART is associated with increased NCD risk factors. Several policy implications can be drawn from our findings. With NCD prevalence rising, population‐based approaches for NCD screening and treatment are urgently needed, in particular among PLHIV, who face an increased risk. Coverage with NCD interventions today is far lower than with HIV treatment 44. Policy makers should learn from the roll‐out of successful HIV interventions when planning NCD interventions.

Integrated approaches to the delivery of HIV and NCD interventions need to be designed, tried and tested in experiments and quasi‐experiments. This could benefit not only PLHIV, but also other population groups at risk for NCDs. While most ART programmes are vertical, such an integrated approach is likely to be more effective as more horizontal chronic disease treatment platforms. This strategy would simultaneously accommodate the change in character of HIV from a deadly infection into a chronic disease. Besides treatment, it is also important to strengthen population‐based health promotion and prevention programmes for NCD risk factors.

The results give empirical support to the sentiment that NCDs will soon be the greatest cause of mortality in sub‐Saharan Africa. This emphasizes the need for comprehensive healthcare programmes that focus on the screening and treatment of both HIV and NCDs, in order to reduce potential mortality rates owed to NCDs.

## Competing interests

We declare no competing interests.

## Authors’ contributions

SV, TB, PG and JWDN conceptualized the study. LC and LB conducted the analysis. LC, LB and SV wrote the first draft of the manuscript. All authors critically reviewed and edited the manuscript.

### Funding

Alexander von Humboldt Foundation.

## Supporting information


**Table SA1.** Data notes
**Table SB1.** Diabetes
**Table SB2.** Mean BMI
**Table SB3.** Overweight
**Table SB4.** Obesity
**Table SB5.** Hypertension (Raised BP)
**Table SB6.** Systolic BP
**Table SC1.** Regression results with year dummies
**Table SC2.** Regression results with quadratic time indicator
**Table SC3.** Regression results without top and bottom 3 percentiles
