# Supplementary material for: HIV, antiretroviral therapy and non‐communicable diseases in sub‐Saharan Africa: empirical evidence from 44 countries over the period 2000 to 2016
Source: J Int AIDS Soc. 2019 Jul 28;22(7):e25364. doi: 10.1002/jia2.25364 (PMC6661400; doi:10.1002/jia2.25364)
Supplement: Supplementary file 1 — Table SA1. Data notes Table SB1. Diabetes Table SB2. Mean BMI Table SB3. Overweight Table SB4. Obesity Table SB5. Hypertension (Raised BP) Table SB6. Systolic BP Table SC1. Regression results with year dummies Table SC2. Regression results with quadratic time indicator Table SC3. Regression results without top and bottom 3 percentiles [file JIA2-22-e25364-s001.docx]

**Appendix S1**

**Table SA1: Data notes**

| Variable | Definition | Source | Coverage |
| --- | --- | --- | --- |
| diabetes | Diabetes prevalence | NCD RisC | 2000 – 2014 |
| raisedBP | Prevalence of raised blood pressure | NCD RisC | 2000 – 2015 |
| sysBP | Mean systolic blood pressure (mmHg) | NCD RisC | 2000 – 2015 |
| hivprev | Percentage of people ages 15-49 who are infected with HIV | World Bank, from UNAIDS | 2000 – 2016 |
| ART | Percentage of total population on antiretroviral therapy (calculated as the proportion of HIV-positive times proportion of HIV-positives on ART) | World Bank, from UNAIDS | 2000 – 2016 |
| meanbmi | Mean body mass index (kg/m^2^) | NCD RisC | 2000 – 2016 |
| overweight | Prevalence of BMI >= 25 kg/m^2^ | NCD RisC | 2000 – 2016 |
| obese | Prevalence of BMI >= 30 kg/m^2^ | NCD RisC | 2000 – 2016 |
| enrol | Total enrolment in primary and secondary education, regardless of age, expressed as a percentage of the population of official primary and secondary education age | World Bank, from UNESCO | 2000 – 2015 |
| loggdp | Gross domestic product divided by midyear population, in constant 2010 U.S. dollars | World Bank | 2000 – 2016 |
| perc2039 | Total 20-39 years old to all population (%) | United Nations | 2000 – 2015^[[1]](#footnote-1)^ |
| perc4059 | Total 40-59 years old to all population (%) | United Nations | 2000 – 2015^7^ |
| perc60 | Total 60 years and older to all population (%) | United Nations | 2000 – 2015^7^ |

**Appendix S2: Regression results with linear time trend**

**Table SB1: Diabetes**

| VARIABLES | 1 | 2 | 3 | 4 | 5 | 6 |
| --- | --- | --- | --- | --- | --- | --- |
|  |  |  |  |  |  |  |
| HIV | 0.089** | 0.097** | 0.074** | 0.063*** | 0.059*** | 0.058*** |
|  | (0.015 - 0.163) | (0.020 - 0.174) | (0.010 - 0.139) | (0.022 - 0.104) | (0.018 - 0.100) | (0.018 - 0.098) |
| ART |  | 0.021* | 0.029*** | 0.029*** | 0.029*** | 0.046*** |
|  |  | (-0.003 - 0.045) | (0.012 - 0.046) | (0.011 - 0.047) | (0.011 - 0.047) | (0.026 - 0.066) |
| BMI |  |  | 0.010*** | 0.010*** | 0.010*** | 0.009*** |
|  |  |  | (0.008 - 0.013) | (0.007 - 0.012) | (0.007 - 0.013) | (0.006 - 0.012) |
| Education |  |  |  | -0.010** | -0.010** | -0.012*** |
|  |  |  |  | (-0.019 - -0.002) | (-0.019 - -0.002) | (-0.021 - -0.003) |
| Income |  |  |  |  | 0.001 | 0.000 |
|  |  |  |  |  | (-0.001 - 0.003) | (-0.002 - 0.002) |
| Age 20-39 |  |  |  |  |  | -0.000 |
|  |  |  |  |  |  | (-0.034 - 0.033) |
| Age 40-59 |  |  |  |  |  | 0.051** |
|  |  |  |  |  |  | (0.009 - 0.094) |
| Age 60+ |  |  |  |  |  | -0.130 |
|  |  |  |  |  |  | (-0.298 - 0.037) |
| Year | 0.001*** | 0.001*** | 0.001*** | 0.001*** | 0.001*** | 0.001*** |
|  | (0.001 - 0.002) | (0.001 - 0.002) | (0.000 - 0.001) | (0.001 - 0.001) | (0.000 - 0.001) | (0.001 - 0.001) |
| Constant | -2.899*** | -2.836*** | -1.373*** | -1.681*** | -1.601*** | -1.726*** |
|  | (-3.185 - -2.613) | (-3.133 - -2.539) | (-1.745 - -1.001) | (-2.080 - -1.282) | (-2.032 - -1.170) | (-2.210 - -1.242) |
|  |  |  |  |  |  |  |
| Observations | 660 | 660 | 660 | 562 | 559 | 559 |
| Number of countries | 44 | 44 | 44 | 42 | 42 | 42 |
| Adjusted R-squared | 0.937 | 0.939 | 0.961 | 0.970 | 0.970 | 0.973 |

Outcome variable: diabetes prevalence in percent; 95% confidence intervals in brackets; * p < 0.1, ** p < 0.05, *** p < 0.01; all models adjusted for fixed effects

**Table SB2: Mean BMI**

| VARIABLES | 1 | 2 | 3 | 4 | 5 |
| --- | --- | --- | --- | --- | --- |
|  |  |  |  |  |  |
| HIV | 2.363 | 2.119 | 1.217 | 1.434 | 0.665 |
|  | (-1.195 - 5.920) | (-0.999 - 5.238) | (-1.617 - 4.051) | (-1.534 - 4.403) | (-1.813 - 3.142) |
| ART |  | -0.613 | -0.089 | -0.220 | 1.229 |
|  |  | (-2.251 - 1.024) | (-1.793 - 1.616) | (-1.961 - 1.522) | (-0.558 - 3.016) |
| Education |  |  | 0.397 | 0.292 | 0.298 |
|  |  |  | (-0.184 - 0.979) | (-0.233 - 0.817) | (-0.231 - 0.828) |
| Income |  |  |  | -0.030 | -0.077 |
|  |  |  |  | (-0.283 - 0.223) | (-0.327 - 0.172) |
| Age 20-39 |  |  |  |  | -2.067* |
|  |  |  |  |  | (-4.343 - 0.209) |
| Age 40-59 |  |  |  |  | 3.951** |
|  |  |  |  |  | (0.223 - 7.679) |
| Age 60+ |  |  |  |  | -2.709 |
|  |  |  |  |  | (-14.449 - 9.030) |
| Year | 0.077*** | 0.078*** | 0.070*** | 0.073*** | 0.074*** |
|  | (0.070 - 0.084) | (0.070 - 0.086) | (0.058 - 0.082) | (0.062 - 0.084) | (0.062 - 0.086) |
| Constant | -132.096*** | -134.160*** | -117.685*** | -124.407*** | -124.831*** |
|  | (-146.026 - -118.165) | (-150.550 - -117.770) | (-140.976 - -94.394) | (-145.844 - -102.969) | (-148.020 - -101.642) |
|  |  |  |  |  |  |
| Observations | 748 | 748 | 582 | 578 | 578 |
| Number of countries | 44 | 44 | 42 | 42 | 42 |
| Adjusted R-squared | 0.933 | 0.934 | 0.933 | 0.938 | 0.942 |

Outcome variable: mean BMI (kg/m^2^); 95% confidence intervals in brackets; * p < 0.1, ** p < 0.05, *** p < 0.01; all models adjusted for fixed effects

**Table SB3: Overweight**

| VARIABLES | 1 | 2 | 3 | 4 | 5 |
| --- | --- | --- | --- | --- | --- |
|  |  |  |  |  |  |
| HIV | 0.117* | 0.139** | 0.137** | 0.142* | 0.111 |
|  | (-0.020 - 0.254) | (0.010 - 0.268) | (0.001 - 0.273) | (-0.000 - 0.284) | (-0.023 - 0.245) |
| ART |  | 0.055* | 0.058 | 0.056 | 0.073 |
|  |  | (-0.010 - 0.120) | (-0.016 - 0.133) | (-0.017 - 0.130) | (-0.037 - 0.182) |
| Education |  |  | 0.014 | 0.012 | 0.009 |
|  |  |  | (-0.012 - 0.040) | (-0.014 - 0.039) | (-0.014 - 0.032) |
| Income |  |  |  | -0.001 | -0.001 |
|  |  |  |  | (-0.010 - 0.008) | (-0.011 - 0.009) |
| Age 20-39 |  |  |  |  | 0.012 |
|  |  |  |  |  | (-0.148 - 0.172) |
| Age 40-59 |  |  |  |  | 0.230** |
|  |  |  |  |  | (0.019 - 0.440) |
| Age 60+ |  |  |  |  | 0.284 |
|  |  |  |  |  | (-0.343 - 0.911) |
| Year | 0.006*** | 0.006*** | 0.005*** | 0.005*** | 0.005*** |
|  | (0.005 - 0.006) | (0.005 - 0.006) | (0.005 - 0.006) | (0.005 - 0.006) | (0.005 - 0.006) |
| Constant | -11.214*** | -11.029*** | -10.393*** | -10.513*** | -10.246*** |
|  | (-11.845 - -10.584) | (-11.657 - -10.401) | (-11.476 - -9.311) | (-11.697 - -9.330) | (-11.217 - -9.274) |
|  |  |  |  |  |  |
| Observations | 748 | 748 | 582 | 578 | 578 |
| Number of countries | 44 | 44 | 42 | 42 | 42 |
| Adjusted R-squared | 0.977 | 0.978 | 0.979 | 0.979 | 0.981 |

Outcome variable: prevalence of BMI>=25kg/m^2^ in percent; 95% confidence intervals in brackets; * p < 0.1, ** p < 0.05, *** p < 0.01; all models adjusted for fixed effects

**Table SB4: Obesity**

| VARIABLES | 1 | 2 | 3 | 4 | 5 |
| --- | --- | --- | --- | --- | --- |
|  |  |  |  |  |  |
| HIV | 0.103 | 0.155** | 0.167* | 0.187** | 0.147** |
|  | (-0.091 - 0.298) | (0.004 - 0.305) | (-0.003 - 0.336) | (0.022 - 0.351) | (0.025 - 0.269) |
| ART |  | 0.130*** | 0.137*** | 0.136*** | 0.140*** |
|  |  | (0.063 - 0.197) | (0.061 - 0.212) | (0.064 - 0.209) | (0.048 - 0.232) |
| Education |  |  | -0.000 | -0.002 | -0.003 |
|  |  |  | (-0.027 - 0.027) | (-0.028 - 0.025) | (-0.023 - 0.017) |
| Income |  |  |  | -0.005 | -0.005 |
|  |  |  |  | (-0.013 - 0.002) | (-0.013 - 0.004) |
| Age 20-39 |  |  |  |  | -0.003 |
|  |  |  |  |  | (-0.144 - 0.138) |
| Age 40-59 |  |  |  |  | 0.234** |
|  |  |  |  |  | (0.056 - 0.413) |
| Age 60+ |  |  |  |  | 0.547** |
|  |  |  |  |  | (0.034 - 1.061) |
| Year | 0.003*** | 0.003*** | 0.003*** | 0.003*** | 0.003*** |
|  | (0.003 - 0.003) | (0.002 - 0.003) | (0.002 - 0.003) | (0.002 - 0.003) | (0.002 - 0.003) |
| Constant | -5.902*** | -5.465*** | -5.267*** | -5.597*** | -5.248*** |
|  | (-6.608 - -5.197) | (-5.980 - -4.949) | (-6.425 - -4.109) | (-6.846 - -4.348) | (-6.134 - -4.363) |
|  |  |  |  |  |  |
| Observations | 748 | 748 | 582 | 578 | 578 |
| Number of countries | 44 | 44 | 42 | 42 | 42 |
| Adjusted R-squared | 0.922 | 0.942 | 0.934 | 0.937 | 0.951 |

Outcome variable: prevalence of BMI>=30kg/m^2^ in percent; 95% confidence intervals in brackets; * p < 0.1, ** p < 0.05, *** p < 0.01; all models adjusted for fixed effects

**Table SB5: Hypertension (Raised BP)**

| VARIABLES | 1 | 2 | 3 | 4 | 5 | 6 |
| --- | --- | --- | --- | --- | --- | --- |
|  |  |  |  |  |  |  |
| HIV | -0.095 | -0.117 | -0.095 | -0.150 | -0.182 | -0.096 |
|  | (-0.330 - 0.141) | (-0.347 - 0.113) | (-0.337 - 0.147) | (-0.422 - 0.122) | (-0.450 - 0.086) | (-0.305 - 0.112) |
| ART |  | -0.056 | -0.064 | -0.009 | -0.008 | -0.090 |
|  |  | (-0.171 - 0.058) | (-0.186 - 0.058) | (-0.132 - 0.114) | (-0.128 - 0.113) | (-0.286 - 0.106) |
| BMI |  |  | -0.010 | -0.007 | -0.004 | 0.003 |
|  |  |  | (-0.029 - 0.008) | (-0.027 - 0.012) | (-0.024 - 0.015) | (-0.019 - 0.024) |
| Education |  |  |  | -0.004 | -0.002 | -0.006 |
|  |  |  |  | (-0.060 - 0.051) | (-0.057 - 0.052) | (-0.049 - 0.037) |
| Income |  |  |  |  | 0.007 | 0.009 |
|  |  |  |  |  | (-0.010 - 0.025) | (-0.010 - 0.028) |
| Age 20-39 |  |  |  |  |  | 0.150 |
|  |  |  |  |  |  | (-0.183 - 0.482) |
| Age 40-59 |  |  |  |  |  | -0.480*** |
|  |  |  |  |  |  | (-0.797 - -0.162) |
| Age 60+ |  |  |  |  |  | -0.612 |
|  |  |  |  |  |  | (-1.747 - 0.523) |
| Year | -0.001*** | -0.001** | 0.000 | 0.000 | -0.000 | -0.001 |
|  | (-0.001 - -0.000) | (-0.001 - -0.000) | (-0.001 - 0.002) | (-0.002 - 0.002) | (-0.002 - 0.001) | (-0.003 - 0.001) |
| Constant | 1.615*** | 1.434*** | 0.021 | 0.329 | 1.146 | 1.624 |
|  | (0.659 - 2.571) | (0.394 - 2.473) | (-2.770 - 2.812) | (-2.661 - 3.318) | (-1.871 - 4.164) | (-1.645 - 4.894) |
|  |  |  |  |  |  |  |
| Observations | 704 | 704 | 704 | 582 | 578 | 578 |
| Number of countries | 44 | 44 | 44 | 42 | 42 | 42 |
| Adjusted R-squared | 0.136 | 0.148 | 0.164 | 0.100 | 0.115 | 0.254 |

Outcome variable: prevalence of raised blood pressure in percent; 95% confidence intervals in brackets; * p < 0.1, ** p < 0.05, *** p < 0.01; all models adjusted for fixed effects

**Table SB6: Systolic BP**

| VARIABLES | 1 | 2 | 3 | 4 | 5 | 6 |
| --- | --- | --- | --- | --- | --- | --- |
|  |  |  |  |  |  |  |
| HIV | -17.015 | -18.563 | -18.075 | -20.982 | -22.919* | -17.385 |
|  | (-39.874 - 5.844) | (-41.493 - 4.366) | (-41.450 - 5.301) | (-46.269 - 4.305) | (-48.762 - 2.924) | (-41.736 - 6.965) |
| ART |  | -3.932 | -4.099 | -1.278 | -1.363 | -8.324 |
|  |  | (-12.654 - 4.790) | (-12.996 - 4.799) | (-10.769 - 8.212) | (-10.801 - 8.075) | (-24.783 - 8.134) |
| BMI |  |  | -0.229 | -0.163 | -0.070 | 0.425 |
|  |  |  | (-1.722 - 1.263) | (-1.724 - 1.398) | (-1.724 - 1.584) | (-1.336 - 2.186) |
| Education |  |  |  | 0.053 | 0.099 | -0.085 |
|  |  |  |  | (-3.953 - 4.060) | (-3.846 - 4.045) | (-3.868 - 3.697) |
| Income |  |  |  |  | 0.502 | 0.694 |
|  |  |  |  |  | (-1.067 - 2.072) | (-1.096 - 2.483) |
| Age 20-39 |  |  |  |  |  | 11.038 |
|  |  |  |  |  |  | (-17.281 - 39.356) |
| Age 40-59 |  |  |  |  |  | -32.407*** |
|  |  |  |  |  |  | (-54.985 - -9.829) |
| Age 60+ |  |  |  |  |  | -23.994 |
|  |  |  |  |  |  | (-120.622 - 72.635) |
| Year | 0.006 | 0.013 | 0.031 | 0.020 | -0.002 | -0.028 |
|  | (-0.033 - 0.046) | (-0.030 - 0.055) | (-0.099 - 0.161) | (-0.110 - 0.150) | (-0.141 - 0.137) | (-0.182 - 0.126) |
| Constant | 117.288*** | 104.648** | 73.273 | 94.204 | 132.070 | 174.603 |
|  | (36.921 - 197.656) | (19.608 - 189.688) | (-156.696 - 303.243) | (-134.923 - 323.331) | (-114.483 - 378.623) | (-94.830 - 444.037) |
|  |  |  |  |  |  |  |
| Observations | 704 | 704 | 704 | 582 | 578 | 578 |
| Number of countries | 44 | 44 | 44 | 42 | 42 | 42 |
| Adjusted R-squared | 0.069 | 0.077 | 0.077 | 0.101 | 0.105 | 0.181 |

Outcome variable: mean systolic blood pressure (mmHg); 95% confidence intervals in brackets; * p < 0.1, ** p < 0.05, *** p < 0.01; all models adjusted for fixed effects

**Appendix S3: Regression results of robustness checks**

**Table SA1: Regression results with year dummies**

| VARIABLES | Diabetes prevalence | Mean BMI | Overweight prevalence | Obesity prevalence | Hypertension prevalence | Mean systolic blood pressure |
| --- | --- | --- | --- | --- | --- | --- |
|  |  |  |  |  |  |  |
| HIV | 0.056*** | 0.915 | 0.110 | 0.146** | -0.101 | -16.736 |
|  | (0.015 - 0.097) | (-1.716 - 3.546) | (-0.026 - 0.245) | (0.021 - 0.271) | (-0.316 - 0.114) | (-41.809 - 8.337) |
| ART | 0.042*** | 1.697* | 0.069 | 0.136*** | -0.095 | -7.033 |
|  | (0.022 - 0.062) | (-0.119 - 3.512) | (-0.044 - 0.182) | (0.042 - 0.231) | (-0.297 - 0.107) | (-24.336 - 10.270) |
| BMI | 0.010*** |  |  |  | 0.004 | 0.074 |
|  | (0.007 - 0.013) |  |  |  | (-0.023 - 0.030) | (-2.011 - 2.159) |
| Education | -0.013*** | 0.426 | 0.008 | -0.004 | -0.007 | 0.262 |
|  | (-0.023 - -0.004) | (-0.100 - 0.952) | (-0.016 - 0.032) | (-0.025 - 0.017) | (-0.053 - 0.039) | (-3.574 - 4.097) |
| Income | 0.000 | -0.070 | -0.001 | -0.005 | 0.009 | 0.682 |
|  | (-0.002 - 0.002) | (-0.315 - 0.175) | (-0.012 - 0.010) | (-0.013 - 0.004) | (-0.010 - 0.028) | (-1.141 - 2.506) |
| Age 20-39 | 0.004 | -2.415** | 0.015 | 0.001 | 0.155 | 9.655 |
|  | (-0.030 - 0.038) | (-4.686 - -0.143) | (-0.149 - 0.179) | (-0.144 - 0.145) | (-0.190 - 0.500) | (-19.590 - 38.900) |
| Age 40-59 | 0.045** | 4.437** | 0.225** | 0.229** | -0.489*** | -30.110*** |
|  | (0.003 - 0.088) | (0.487 - 8.386) | (0.012 - 0.437) | (0.049 - 0.410) | (-0.807 - -0.171) | (-52.044 - -8.176) |
| Age 60+ | -0.136 | -1.681 | 0.283 | 0.543** | -0.619 | -23.535 |
|  | (-0.317 - 0.046) | (-14.435 - 11.072) | (-0.357 - 0.923) | (0.010 - 1.076) | (-1.762 - 0.524) | (-123.058 - 75.987) |
| Constant | -0.177*** | 22.703*** | 0.163*** | 0.019 | 0.208 | 125.798*** |
|  | (-0.251 - -0.103) | (20.680 - 24.725) | (0.067 - 0.259) | (-0.059 - 0.098) | (-0.390 - 0.805) | (77.949 - 173.646) |
|  |  |  |  |  |  |  |
| Observations | 559 | 578 | 578 | 578 | 578 | 578 |
| Number of iso3 | 42 | 42 | 42 | 42 | 42 | 42 |
| Adjusted R-squared | 0.974 | 0.951 | 0.981 | 0.950 | 0.238 | 0.173 |

Results of linear regressions; 95% confidence intervals in brackets; * p < 0.1, ** p < 0.05, *** p < 0.01; all models adjusted for fixed effects; all models include year dummies; outcome variables: diabetes prevalence in percent; country-level mean BMI (kg/m^2^); overweight as prevalence of BMI>=25kg/m^2^ in percent; overweight as prevalence of BMI>=30kg/m^2^ in percent; prevalence of raised blood pressure in percent; country-level mean systolic blood pressure (mm Hg).

**Table SC2: Regression results with quadratic time indicator**

| VARIABLES | Diabetes prevalence | Mean BMI | Overweight prevalence | Obesity prevalence | Hypertension prevalence | Mean systolic blood pressure |
| --- | --- | --- | --- | --- | --- | --- |
|  |  |  |  |  |  |  |
| HIV | 0.056*** | 0.900 | 0.109 | 0.145** | -0.099 | -16.681 |
|  | (0.016 - 0.097) | (-1.673 - 3.473) | (-0.024 - 0.242) | (0.022 - 0.268) | (-0.309 - 0.111) | (-41.198 - 7.837) |
| ART | 0.042*** | 1.696* | 0.069 | 0.136*** | -0.094 | -6.958 |
|  | (0.022 - 0.062) | (-0.095 - 3.487) | (-0.044 - 0.181) | (0.042 - 0.229) | (-0.295 - 0.107) | (-24.080 - 10.164) |
| BMI | 0.010*** |  |  |  | 0.004 | 0.076 |
|  | (0.007 - 0.013) |  |  |  | (-0.022 - 0.030) | (-1.994 - 2.146) |
| Education | -0.013*** | 0.423 | 0.008 | -0.004 | -0.007 | 0.269 |
|  | (-0.023 - -0.004) | (-0.092 - 0.939) | (-0.016 - 0.031) | (-0.025 - 0.016) | (-0.052 - 0.038) | (-3.493 - 4.031) |
| Income | 0.000 | -0.070 | -0.001 | -0.005 | 0.009 | 0.680 |
|  | (-0.002 - 0.002) | (-0.309 - 0.168) | (-0.011 - 0.009) | (-0.013 - 0.004) | (-0.010 - 0.028) | (-1.105 - 2.465) |
| Age 20-39 | 0.004 | -2.422** | 0.015 | 0.001 | 0.154 | 9.604 |
|  | (-0.030 - 0.037) | (-4.680 - -0.164) | (-0.147 - 0.177) | (-0.142 - 0.143) | (-0.186 - 0.495) | (-19.300 - 38.507) |
| Age 40-59 | 0.045** | 4.447** | 0.226** | 0.229** | -0.487*** | -30.033*** |
|  | (0.002 - 0.087) | (0.537 - 8.356) | (0.015 - 0.436) | (0.051 - 0.408) | (-0.802 - -0.173) | (-51.806 - -8.261) |
| Age 60+ | -0.136 | -1.758 | 0.276 | 0.538** | -0.615 | -23.031 |
|  | (-0.316 - 0.043) | (-14.297 - 10.781) | (-0.358 - 0.910) | (0.012 - 1.065) | (-1.739 - 0.510) | (-121.048 - 74.986) |
| time | 0.000 | 0.140*** | 0.005*** | 0.002*** | -0.001 | 0.131 |
|  | (-0.000 - 0.001) | (0.117 - 0.164) | (0.004 - 0.006) | (0.001 - 0.003) | (-0.005 - 0.003) | (-0.204 - 0.465) |
| time squared | 0.000** | -0.002*** | 0.000 | 0.000* | 0.000 | -0.004 |
|  | (0.000 - 0.000) | (-0.003 - -0.001) | (-0.000 - 0.000) | (-0.000 - 0.000) | (-0.000 - 0.000) | (-0.009 - 0.002) |
| Constant | -0.182*** | 21.396*** | 0.111** | -0.005 | 0.220 | 124.761*** |
|  | (-0.251 - -0.112) | (19.504 - 23.288) | (0.020 - 0.201) | (-0.079 - 0.069) | (-0.336 - 0.776) | (80.200 - 169.323) |
|  |  |  |  |  |  |  |
| Observations | 559 | 578 | 578 | 578 | 578 | 578 |
| Number of iso3 | 42 | 42 | 42 | 42 | 42 | 42 |
| Adjusted R-squared | 0.974 | 0.952 | 0.981 | 0.951 | 0.254 | 0.191 |

Results of linear regressions; 95% confidence intervals in brackets; * p < 0.1, ** p < 0.05, *** p < 0.01; all models adjusted for fixed effects; outcome variables: diabetes prevalence in percent; country-level mean BMI (kg/m^2^); overweight as prevalence of BMI>=25kg/m^2^ in percent; overweight as prevalence of BMI>=30kg/m^2^ in percent; prevalence of raised blood pressure in percent; country-level mean systolic blood pressure (mm Hg).

**Table SC3: Regression results without top and bottom 3 percentiles**

| VARIABLES | Diabetes prevalence | Mean BMI | Overweight prevalence | Obesity prevalence | Hypertension prevalence | Mean systolic blood pressure |
| --- | --- | --- | --- | --- | --- | --- |
|  |  |  |  |  |  |  |
| HIV | 0.044** | 0.531 | 0.073 | 0.084 | -0.127 | -16.689 |
|  | (0.004 - 0.085) | (-2.034 - 3.096) | (-0.090 - 0.236) | (-0.058 - 0.225) | (-0.334 - 0.080) | (-40.919 - 7.540) |
| ART | 0.032** | 0.004 | 0.014 | 0.087* | -0.110 | -8.915 |
|  | (0.008 - 0.056) | (-1.704 - 1.711) | (-0.093 - 0.122) | (-0.009 - 0.183) | (-0.298 - 0.077) | (-25.330 - 7.500) |
| BMI | 0.010*** |  |  |  | 0.001 | 0.468 |
|  | (0.008 - 0.013) |  |  |  | (-0.019 - 0.021) | (-1.328 - 2.263) |
| Education | -0.013*** | 0.266 | 0.010 | 0.000 | 0.004 | 0.223 |
|  | (-0.021 - -0.005) | (-0.270 - 0.801) | (-0.015 - 0.035) | (-0.021 - 0.022) | (-0.037 - 0.044) | (-3.577 - 4.023) |
| Income | 0.001 | -0.008 | 0.001 | -0.003 | 0.012 | 0.748 |
|  | (-0.001 - 0.003) | (-0.240 - 0.223) | (-0.008 - 0.010) | (-0.009 - 0.004) | (-0.005 - 0.029) | (-0.967 - 2.462) |
| Age 20-39 | 0.029 | -1.428 | 0.091 | 0.071 | 0.159 | 12.710 |
|  | (-0.018 - 0.076) | (-3.660 - 0.804) | (-0.093 - 0.274) | (-0.105 - 0.247) | (-0.162 - 0.480) | (-19.322 - 44.741) |
| Age 40-59 | 0.030 | 3.074 | 0.158 | 0.170** | -0.367* | -30.384*** |
|  | (-0.016 - 0.076) | (-0.843 - 6.991) | (-0.059 - 0.375) | (0.003 - 0.337) | (-0.757 - 0.023) | (-52.951 - -7.816) |
| Age 60+ | -0.232*** | 2.058 | 0.208 | 0.285 | -0.252 | -18.891 |
|  | (-0.395 - -0.070) | (-12.340 - 16.457) | (-0.602 - 1.018) | (-0.273 - 0.844) | (-1.382 - 0.877) | (-111.218 - 73.436) |
| Year | 0.001*** | 0.074*** | 0.005*** | 0.003*** | -0.001 | -0.042 |
|  | (0.000 - 0.001) | (0.062 - 0.087) | (0.005 - 0.006) | (0.002 - 0.003) | (-0.003 - 0.001) | (-0.212 - 0.128) |
| Constant | -1.583*** | -126.834*** | -10.168*** | -5.063*** | 1.891 | 199.515 |
|  | (-2.052 - -1.114) | (-150.210 - -103.457) | (-11.302 - -9.035) | (-6.042 - -4.083) | (-1.298 - 5.081) | (-99.189 - 498.219) |
|  |  |  |  |  |  |  |
| Observations | 533 | 542 | 540 | 540 | 547 | 544 |
| Number of iso3 | 42 | 42 | 41 | 41 | 42 | 40 |
| Adjusted R-squared | 0.976 | 0.946 | 0.982 | 0.956 | 0.209 | 0.175 |

Results of linear regressions; 95% confidence intervals in brackets; * p < 0.1, ** p < 0.05, *** p < 0.01; all models adjusted for fixed effects; trimmed top and bottom 3 percentiles; outcome variables: diabetes prevalence in percent; country-level mean BMI (kg/m^2^); overweight as prevalence of BMI>=25kg/m^2^ in percent; overweight as prevalence of BMI>=30kg/m^2^ in percent; prevalence of raised blood pressure in percent; country-level mean systolic blood pressure (mm Hg).

1. Data given every 5 years [↑](#footnote-ref-1)
